# Benzyl­ammonium hepta­noate

**DOI:** 10.1107/S1600536813009574

**Published:** 2013-04-20

**Authors:** Mary H. Wood, Stuart M. Clarke

**Affiliations:** a BP Institute and Department of Chemistry, University of Cambridge, Cambridge, England

## Abstract

The title 1:1 stoichiometric salt, C_7_H_10_N^+^·C_7_H_13_O_2_^−^, is formed by proton transfer between hepta­noic acid and benzyl­amine. This combination contrasts to the recently published 2:1 acid–amine adduct of cation, anion and neutral acid molecule from the same components [Wood & Clarke (2013 ▶). *Acta Cryst*. E**69**, o346–o347]. There are N—H⋯O hydrogen bonds of moderate strength in the structure [the most important graph-set motifs are *R* ^2^_4_(8) and *R* ^4^_4_(12)], as well as weak C—H⋯O inter­actions.

## Related literature

For spectroscopic studies of acid–amine complexes, see: Kohler *et al.* (1981 ▶); Karlsson *et al.* (2000 ▶); Paivarinta *et al.* (2000 ▶); Smith *et al.* (2001 ▶, 2002 ▶). For recent diffraction studies of acid–amine complexes, see: Jefferson *et al.* (2011 ▶); Sun *et al.* (2011 ▶); Wood & Clarke (2012*a* ▶,*b* ▶, 2013 ▶). For the categorization of hydrogen bonds, see Gilli & Gilli (2009 ▶). For graph-set motifs, see Etter *et al.* (1990 ▶).
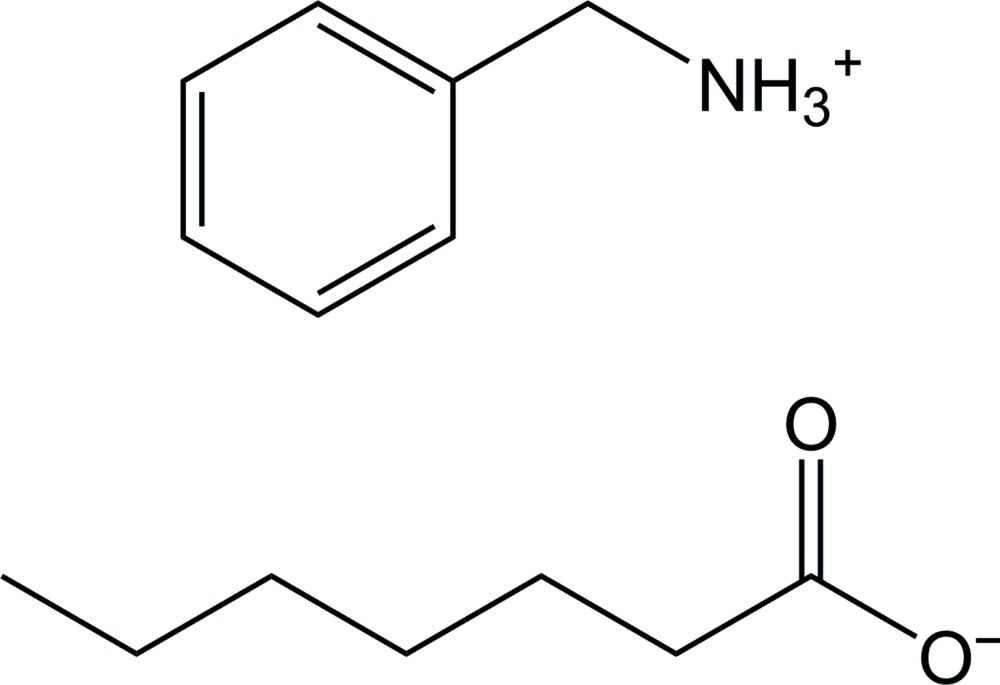


## Experimental

### 

#### Crystal data

C_7_H_10_N^+^·C_7_H_13_O_2_^−^
*M* *_r_* = 237.33
Triclinic, 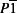

*a* = 5.7379 (2) Å
*b* = 7.7338 (3) Å
*c* = 17.1670 (7) Å
α = 97.887 (2)°
β = 92.864 (2)°
γ = 107.340 (2)°
*V* = 716.96 (5) Å^3^
*Z* = 2
Mo *K*α radiation
μ = 0.07 mm^−1^
*T* = 180 K
0.46 × 0.07 × 0.05 mm

#### Data collection

Nonius KappaCCD diffractometer
Absorption correction: multi-scan (*SORTAV*; Blessing, 1995 ▶) *T*_min_ = 0.874, *T*_max_ = 0.999
8415 measured reflections
3253 independent reflections
2308 reflections with *I* > 2σ(*I*)
*R*_int_ = 0.043

#### Refinement

*R*[*F* ^2^ > 2σ(*F* ^2^)] = 0.054
*wR*(*F* ^2^) = 0.141
*S* = 1.03
3253 reflections
164 parameters
H atoms treated by a mixture of independent and constrained refinement
Δρ_max_ = 0.32 e Å^−3^
Δρ_min_ = −0.19 e Å^−3^

### 

Data collection: *COLLECT* (Nonius, 1998 ▶); cell refinement: *SCALEPACK* (Otwinowski & Minor 1997 ▶); data reduction: *DENZO* (Otwinowski & Minor, 1997 ▶) and *SCALEPACK*; program(s) used to solve structure: *SIR92* (Altomare *et al.* , 1993 ▶); program(s) used to refine structure: *SHELXL97* (Sheldrick, 2008 ▶); molecular graphics: *Mercury* (Macrae *et al.*, 2008 ▶); software used to prepare material for publication: *SHELXL97*.

## Supplementary Material

Crystal structure: contains datablock(s) I, global. DOI: 10.1107/S1600536813009574/fb2281sup1.cif

Structure factors: contains datablock(s) I. DOI: 10.1107/S1600536813009574/fb2281Isup2.hkl

Supplementary material file. DOI: 10.1107/S1600536813009574/fb2281Isup3.cml

Additional supplementary materials:  crystallographic information; 3D view; checkCIF report

## Figures and Tables

**Table 1:** Hydrogen-bond geometry (Å, °)

*D*—H⋯*A*	*D*—H	H⋯*A*	*D*⋯*A*	*D*—H⋯*A*
N1—H1*A*⋯O2	0.99 (2)	1.80 (2)	2.7802 (17)	168.7 (19)
N1—H1*B*⋯O1^i^	0.98 (2)	1.73 (2)	2.6993 (17)	169 (2)
N1—H1*C*⋯O2^ii^	1.00 (2)	1.87 (2)	2.8590 (18)	167 (2)
C1—H1*E*⋯O1^iii^	0.99	2.40	3.280 (2)	148
C7—H7⋯O2	0.95	2.52	3.3528 (19)	146

Symmetry codes: (i) 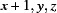; (ii) 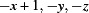; (iii) 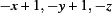.

**Fig. 1.**
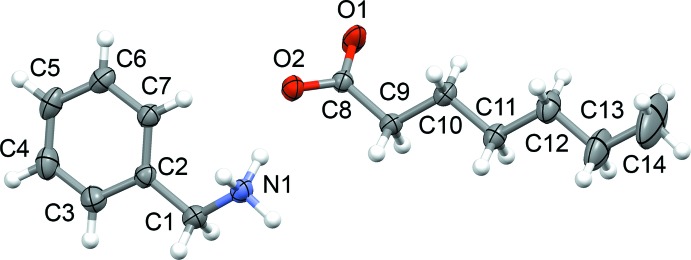
Title molecules with atom labels. The displacement ellipsoids are drawn at the 50% probability level for non-H atoms. H atoms are shown as spheres of arbitrary size.

**Fig. 2.**
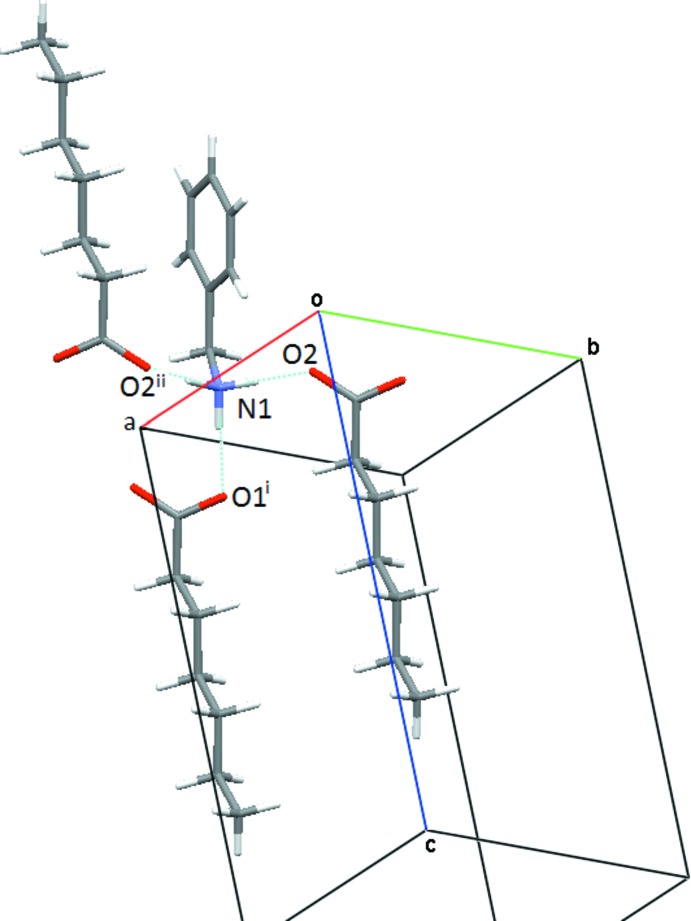
Hydrogen bonding around the ammonium cation. The symmetry codes: i: 1+x, y, z; ii: 1-x, -y, -z.

**Fig. 3.**
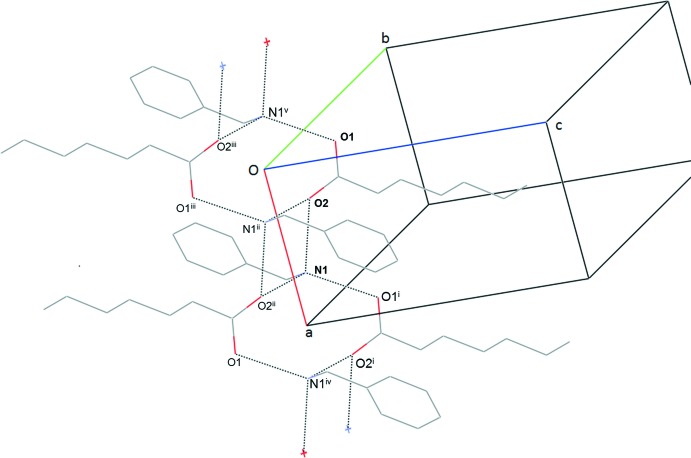
A section from the hydrogen bond pattern in the title structure. The symmetry codes: i: 1+x, y, z; ii: 1-x, -y, -z; iii: -x, -y, -z; iv: 2-x, -y, -z; v: -1+x, y, z.

## supplementary crystallographic information

### Comment

There exist in the literature a number of examples of complexes formed between alkyl amines and carboxylic acids. These have been identified by a number of experimental methods, such as NMR and IR spectroscopy (*e. g.* Karlsson *et al.*, 2000; Paivarinta *et al.* (2000); Smith *et al.* (2001, 2002)). Unfortunately, the atomic details of the materials and hence the nature of the bonding that can be obtained from single-crystal diffraction has only been determined in a few cases. This is partly due to the challenges in growing crystals large enough to be suitable for single-crystal diffraction. Some crystals of sufficient size have been grown (*e. g.* Jefferson *et al.* (2011); Wood & Clarke, 2012*a*, 2012*b*). Of the stoichiometic combinations reported to date, the majority have been 1:1 complexes, though some 2:1 and even 3:1 examples have been reported by calorimetry and NMR spectroscopy, generally when the environment is acid-rich (*e. g.* Kohler *et al.*, 1981; Sun *et al.*, 2011), and very recently using single-crystal diffraction (Wood & Clarke, 2013).

Here we report growth of a suitable crystal for single-crystal X-ray diffraction of a 1:1 complex formed between heptanoic acid and benzylamine (Figs. 1 and 2). This work follows a previous publication (Wood & Clarke, 2013) in which an acid-rich 2:1 complex formed between these two species is described. In the previous work, one acid molecule donates its proton to the amine group and one acid group retains its proton. The hydrogen bonding extends across both the ions and the neutral acid molecule.

As described below, the sample of the present study was grown by vapour phase condensation. Each preparation resulted in a batch of several crystals. The crystal used in this present study was taken from a different region of the same batch of samples as for the 2:1 combination (Wood & Clarke, 2013). We attribute the combination of compositions to the concentration gradients across the vapour streams in the preparation.

In the crystal structure of the 1:1 complex (Figs. 1 and 2), each acid anion is involved in N-H···O hydrogen bonds of moderate strength (Gilli & Gilli, 2009) with three surrounding amine molecules (Table 1; Fig. 2), and *vice versa* each benzylammonium group donates its hydrogen to three different heptanoate molecules. The most important graph set motifs are *R*^2^_4_(8) (Etter *et al.*, 1990) - see Fig.3. (In this motif the donated atoms are H1a and H1c and the acceptors are the O2 atoms.) The other important motif is *R*^4^_4_(12) with donated hydrogens H1b and H1c and accepting oxygens O1 and O2 (Fig. 3.). Moreover, there are also weak C-H···O interactions present in the structure (Table 1).

### Experimental

Benzylamine and heptanoic acid (purities 99.7% and 99.8% respectively, determined by titration and gas chromatography) were purchased from Sigma Aldrich and used without further purification. A small volume of amine (approximately 1 ml) was placed into a small vial that was itself placed within a larger vial containing a similar volume of the acid, and left in an inert atmosphere. Extensive crystal growth was observed after a few weeks, particularly on a polypropylene surface included in the vial to encourage nucleation.

### Refinement

All the hydrogens were discernible in the difference electron density map. Nevertheless, the hydrogens attached to the C atoms were situated in the idealized positions and refined under these constraints: C_aryl_-H_aryl_=0.95, C_methyl_-H_methyl_=0.98, C_methylene_-H_methylene_=0.99 Å. *U*_eq_(H_aryl_)=1.2*U*_iso_(C_aryl_), *U*_eq_(H_methylene_)=1.2*U*_iso_(C_methylene_), *U*_eq_(H_methyl_)=1.5*U*_iso_(C_methyl_). The positional parameters of the hydrogens attached to N of the benzylammonium cation were freely refined while their *U*_eq_(H_N_)=1.5*U*_iso_(H_N_).

### Crystal data

**Table:**

C_7_H_10_N^+^·C_7_H_13_O_2_^−^	*Z* = 2
*M**_r_* = 237.33	*F*(000) = 260
Triclinic, *P*1	*D*_x_ = 1.099 Mg m^−^^3^
Hall symbol: -P 1	Mo *K*α radiation, λ = 0.71073 Å
*a* = 5.7379 (2) Å	Cell parameters from 9084 reflections
*b* = 7.7338 (3) Å	θ = 1.0–27.5°
*c* = 17.1670 (7) Å	µ = 0.07 mm^−^^1^
α = 97.887 (2)°	*T* = 180 K
β = 92.864 (2)°	Needle, colourless
γ = 107.340 (2)°	0.46 × 0.07 × 0.05 mm
*V* = 716.96 (5) Å^3^	

### Data collection

**Table:**

Nonius KappaCCD diffractometer	3253 independent reflections
Radiation source: fine-focus sealed tube	2308 reflections with *I* > 2σ(*I*)
Graphite monochromator	*R*_int_ = 0.043
ω and φ scans	θ_max_ = 27.5°, θ_min_ = 3.6°
Absorption correction: multi-scan (*SORTAV*; Blessing, 1995)	*h* = −7→7
*T*_min_ = 0.874, *T*_max_ = 0.999	*k* = −10→9
8415 measured reflections	*l* = −22→22

### Refinement

**Table:**

Refinement on *F*^2^	Primary atom site location: structure-invariant direct methods
Least-squares matrix: full	Secondary atom site location: difference Fourier map
*R*[*F*^2^ > 2σ(*F*^2^)] = 0.054	Hydrogen site location: difference Fourier map
*wR*(*F*^2^) = 0.141	H atoms treated by a mixture of independent and constrained refinement
*S* = 1.03	*w* = 1/[σ^2^(*F*_o_^2^) + (0.0544*P*)^2^ + 0.2301*P*] where *P* = (*F*_o_^2^ + 2*F*_c_^2^)/3
3253 reflections	(Δ/σ)_max_ < 0.001
164 parameters	Δρ_max_ = 0.32 e Å^−^^3^
0 restraints	Δρ_min_ = −0.19 e Å^−^^3^
82 constraints	

### Special details

**Table:**

Geometry. All e.s.d.'s (except the e.s.d. in the dihedral angle between two l.s. planes) are estimated using the full covariance matrix. The cell e.s.d.'s are taken into account individually in the estimation of e.s.d.'s in distances, angles and torsion angles; correlations between e.s.d.'s in cell parameters are only used when they are defined by crystal symmetry. An approximate (isotropic) treatment of cell e.s.d.'s is used for estimating e.s.d.'s involving l.s. planes.
Refinement. Refinement of *F*^2^ against ALL reflections. The weighted *R*-factor *wR* and goodness of fit *S* are based on *F*^2^, conventional *R*-factors *R* are based on *F*, with *F* set to zero for negative *F*^2^. The threshold expression of *F*^2^ > σ(*F*^2^) is used only for calculating *R*-factors(gt) *etc*. and is not relevant to the choice of reflections for refinement. *R*-factors based on *F*^2^ are statistically about twice as large as those based on *F*, and *R*- factors based on ALL data will be even larger.

### Fractional atomic coordinates and isotropic or equivalent isotropic displacement parameters (Å^2^)

**Table:**

	*x*	*y*	*z*	*U*_iso_*/*U*_eq_	
N1	0.7763 (2)	0.1651 (2)	−0.04200 (8)	0.0352 (3)	
H1A	0.630 (4)	0.195 (3)	−0.0239 (11)	0.053*	
H1B	0.919 (4)	0.247 (3)	−0.0072 (12)	0.053*	
H1C	0.752 (4)	0.033 (3)	−0.0380 (11)	0.053*	
C1	0.8129 (3)	0.1865 (2)	−0.12452 (9)	0.0363 (4)	
H1D	0.9629	0.1556	−0.1381	0.044*	
H1E	0.8402	0.3168	−0.1298	0.044*	
C2	0.6010 (3)	0.06878 (19)	−0.18296 (9)	0.0300 (3)	
C3	0.6366 (3)	0.0514 (2)	−0.26288 (10)	0.0407 (4)	
H3	0.7927	0.1102	−0.2790	0.049*	
C4	0.4468 (4)	−0.0507 (3)	−0.31918 (10)	0.0478 (5)	
H4	0.4734	−0.0608	−0.3736	0.057*	
C5	0.2196 (3)	−0.1378 (2)	−0.29682 (10)	0.0448 (4)	
H5	0.0898	−0.2079	−0.3356	0.054*	
C6	0.1819 (3)	−0.1225 (2)	−0.21798 (10)	0.0379 (4)	
H6	0.0259	−0.1830	−0.2022	0.045*	
C7	0.3712 (3)	−0.0191 (2)	−0.16130 (9)	0.0327 (4)	
H7	0.3430	−0.0084	−0.1070	0.039*	
O1	0.1339 (2)	0.37947 (17)	0.06866 (7)	0.0499 (4)	
O2	0.35258 (19)	0.20902 (14)	0.01788 (6)	0.0345 (3)	
C8	0.3307 (3)	0.34204 (19)	0.06561 (8)	0.0274 (3)	
C9	0.5477 (3)	0.4606 (2)	0.12239 (8)	0.0297 (3)	
H9A	0.5971	0.5868	0.1097	0.036*	
H9B	0.6878	0.4119	0.1154	0.036*	
C10	0.4899 (3)	0.4671 (2)	0.20864 (8)	0.0305 (3)	
H10A	0.3462	0.5117	0.2151	0.037*	
H10B	0.4458	0.3413	0.2217	0.037*	
C11	0.7043 (3)	0.5911 (2)	0.26616 (9)	0.0346 (4)	
H11A	0.8487	0.5476	0.2590	0.042*	
H11B	0.7467	0.7172	0.2535	0.042*	
C12	0.6502 (4)	0.5962 (2)	0.35193 (10)	0.0472 (5)	
H12A	0.6043	0.4697	0.3643	0.057*	
H12B	0.5081	0.6423	0.3594	0.057*	
C13	0.8663 (5)	0.7167 (3)	0.40931 (11)	0.0747 (7)	
H13A	1.0069	0.6686	0.4025	0.090*	
H13B	0.9147	0.8422	0.3958	0.090*	
C14	0.8143 (8)	0.7273 (5)	0.49474 (15)	0.1389 (17)	
H14A	0.9597	0.8085	0.5281	0.208*	
H14B	0.7732	0.6044	0.5094	0.208*	
H14C	0.6761	0.7758	0.5023	0.208*	

### Atomic displacement parameters (Å^2^)

**Table:**

	*U*^11^	*U*^22^	*U*^33^	*U*^12^	*U*^13^	*U*^23^
N1	0.0267 (7)	0.0391 (8)	0.0357 (7)	0.0102 (6)	−0.0036 (6)	−0.0059 (6)
C1	0.0289 (8)	0.0376 (8)	0.0371 (8)	0.0054 (7)	0.0018 (7)	−0.0003 (7)
C2	0.0310 (8)	0.0260 (7)	0.0320 (8)	0.0096 (6)	0.0007 (6)	−0.0001 (6)
C3	0.0426 (9)	0.0408 (9)	0.0362 (9)	0.0095 (8)	0.0054 (7)	0.0040 (7)
C4	0.0623 (12)	0.0500 (10)	0.0280 (8)	0.0155 (9)	−0.0009 (8)	0.0016 (7)
C5	0.0487 (10)	0.0405 (9)	0.0379 (9)	0.0101 (8)	−0.0157 (8)	−0.0032 (7)
C6	0.0330 (8)	0.0344 (8)	0.0428 (9)	0.0075 (7)	−0.0044 (7)	0.0033 (7)
C7	0.0307 (8)	0.0337 (8)	0.0314 (8)	0.0087 (6)	−0.0008 (6)	0.0014 (6)
O1	0.0302 (6)	0.0555 (8)	0.0574 (8)	0.0210 (6)	−0.0126 (5)	−0.0247 (6)
O2	0.0326 (6)	0.0328 (6)	0.0359 (6)	0.0119 (5)	0.0001 (5)	−0.0052 (5)
C8	0.0275 (7)	0.0272 (7)	0.0264 (7)	0.0079 (6)	0.0011 (6)	0.0022 (6)
C9	0.0250 (7)	0.0309 (8)	0.0305 (8)	0.0066 (6)	0.0003 (6)	0.0012 (6)
C10	0.0293 (7)	0.0282 (7)	0.0309 (8)	0.0060 (6)	−0.0010 (6)	0.0020 (6)
C11	0.0363 (8)	0.0321 (8)	0.0306 (8)	0.0059 (7)	−0.0044 (7)	0.0015 (6)
C12	0.0616 (11)	0.0419 (10)	0.0307 (9)	0.0070 (9)	−0.0012 (8)	0.0036 (7)
C13	0.0991 (19)	0.0672 (14)	0.0341 (10)	−0.0026 (13)	−0.0178 (11)	0.0004 (10)
C14	0.196 (4)	0.128 (3)	0.0330 (13)	−0.031 (3)	−0.0145 (17)	−0.0003 (15)

### Geometric parameters (Å, º)

**Table:**

N1—C1	1.467 (2)	C8—C9	1.5148 (19)
N1—H1A	0.99 (2)	C9—C10	1.531 (2)
N1—H1B	0.98 (2)	C9—H9A	0.9900
N1—H1C	1.00 (2)	C9—H9B	0.9900
C1—C2	1.510 (2)	C10—C11	1.524 (2)
C1—H1D	0.9900	C10—H10A	0.9900
C1—H1E	0.9900	C10—H10B	0.9900
C2—C7	1.386 (2)	C11—C12	1.518 (2)
C2—C3	1.391 (2)	C11—H11A	0.9900
C3—C4	1.384 (2)	C11—H11B	0.9900
C3—H3	0.9500	C12—C13	1.521 (3)
C4—C5	1.378 (3)	C12—H12A	0.9900
C4—H4	0.9500	C12—H12B	0.9900
C5—C6	1.376 (2)	C13—C14	1.507 (3)
C5—H5	0.9500	C13—H13A	0.9900
C6—C7	1.389 (2)	C13—H13B	0.9900
C6—H6	0.9500	C14—H14A	0.9800
C7—H7	0.9500	C14—H14B	0.9800
O1—C8	1.2486 (17)	C14—H14C	0.9800
O2—C8	1.2653 (17)		
			
C1—N1—H1A	113.5 (11)	C10—C9—H9A	109.1
C1—N1—H1B	109.7 (11)	C8—C9—H9B	109.1
H1A—N1—H1B	107.3 (15)	C10—C9—H9B	109.1
C1—N1—H1C	107.3 (11)	H9A—C9—H9B	107.9
H1A—N1—H1C	107.5 (16)	C11—C10—C9	112.75 (12)
H1B—N1—H1C	111.6 (16)	C11—C10—H10A	109.0
N1—C1—C2	114.05 (13)	C9—C10—H10A	109.0
N1—C1—H1D	108.7	C11—C10—H10B	109.0
C2—C1—H1D	108.7	C9—C10—H10B	109.0
N1—C1—H1E	108.7	H10A—C10—H10B	107.8
C2—C1—H1E	108.7	C12—C11—C10	113.17 (14)
H1D—C1—H1E	107.6	C12—C11—H11A	108.9
C7—C2—C3	118.30 (14)	C10—C11—H11A	108.9
C7—C2—C1	123.43 (13)	C12—C11—H11B	108.9
C3—C2—C1	118.25 (14)	C10—C11—H11B	108.9
C4—C3—C2	120.71 (16)	H11A—C11—H11B	107.8
C4—C3—H3	119.6	C11—C12—C13	113.06 (16)
C2—C3—H3	119.6	C11—C12—H12A	109.0
C5—C4—C3	120.39 (16)	C13—C12—H12A	109.0
C5—C4—H4	119.8	C11—C12—H12B	109.0
C3—C4—H4	119.8	C13—C12—H12B	109.0
C6—C5—C4	119.57 (15)	H12A—C12—H12B	107.8
C6—C5—H5	120.2	C14—C13—C12	113.9 (2)
C4—C5—H5	120.2	C14—C13—H13A	108.8
C5—C6—C7	120.24 (16)	C12—C13—H13A	108.8
C5—C6—H6	119.9	C14—C13—H13B	108.8
C7—C6—H6	119.9	C12—C13—H13B	108.8
C2—C7—C6	120.78 (15)	H13A—C13—H13B	107.7
C2—C7—H7	119.6	C13—C14—H14A	109.5
C6—C7—H7	119.6	C13—C14—H14B	109.5
O1—C8—O2	122.39 (13)	H14A—C14—H14B	109.5
O1—C8—C9	117.80 (12)	C13—C14—H14C	109.5
O2—C8—C9	119.80 (12)	H14A—C14—H14C	109.5
C8—C9—C10	112.28 (12)	H14B—C14—H14C	109.5
C8—C9—H9A	109.1		

### Hydrogen-bond geometry (Å, º)

**Table:**

*D*—H···*A*	*D*—H	H···*A*	*D*···*A*	*D*—H···*A*
N1—H1*A*···O2	0.99 (2)	1.80 (2)	2.7802 (17)	168.7 (19)
N1—H1*B*···O1^i^	0.98 (2)	1.73 (2)	2.6993 (17)	169 (2)
N1—H1*C*···O2^ii^	1.00 (2)	1.87 (2)	2.8590 (18)	167 (2)
C1—H1*E*···O1^iii^	0.99	2.40	3.280 (2)	148
C7—H7···O2	0.95	2.52	3.3528 (19)	146

Symmetry codes: (i) *x*+1, *y*, *z*; (ii) −*x*+1, −*y*, −*z*; (iii) −*x*+1, −*y*+1, −*z*.
